# Effect of Ag^+^ and Cd^2+^ Elicitation on Polyphenol Production in Shoot Culture of *Dracocephalum ruyschiana* L.

**DOI:** 10.3390/molecules29225263

**Published:** 2024-11-07

**Authors:** Izabela Weremczuk-Jeżyna, Jan Gomulski, Anna K. Kiss, Izabela Grzegorczyk-Karolak

**Affiliations:** 1 Department of Biology and Pharmaceutical Botany, Medical University of Lodz, 90-151 Lodz, Poland; jan.gomulski@umed.lodz.pl (J.G.); izabela.grzegorczyk@umed.lodz.pl (I.G.-K.); 2 Department of Pharmaceutical Biology, Medical University of Warsaw, 02-097 Warsaw, Poland; anna.kiss@wum.edu.pl

**Keywords:** abiotic elicitors, apigenin glycosides, antioxidant activity, antioxidant enzymes, heavy metals, rosmarinic acid

## Abstract

Abiotic elicitation with heavy metals has demonstrated considerable potential to stimulate the production of industrially important secondary metabolites in plant in vitro cultures. The present study investigates the effect of exogenous silver nitrate and cadmium chloride supplementation on flavonoid and phenolic acid production, as well as other indicators of oxidative stress, in shoot cultures of *Dracocephalum ruyschiana* L. Owing to the presence of bioactive polyphenolic compounds, this Mongolian medicinal plant is traditionally used as an anti-inflammatory, antibacterial and antipyretic agent. The shoots were cultured for three weeks, and then, cadmium (Cd^2+^) and silver (Ag^+^) ions (50 or 100 µM) were added to the medium. The maximum proliferation rate was observed in the presence of 100 µM Ag^+^ (almost 5), the highest chlorophyll content in the presence of 100 µM Cd^2+^ (0.6 mg/g FW) and the highest biomass was observed with both these treatments (73.4–75.7 g FW and 7.53–7.72 g DW). UPLC-PDA-ESI-MS analysis revealed four phenolic acids and five flavonoid derivatives in the hydromethanolic extract of *D. ruyschiana* shoots. All treatments stimulated the production of rosmarinic acid (RA), which was the dominant compound in the analyzed culture; the highest level of RA, i.e., about three times higher than the control, was noted in shoots exposed to 50 µM Cd^2+^ (14.72 mg/g DW), whereas the level of most flavonoids in the culture increased most significantly when exposed to Cd^2+^ at a concentration of 100 µM. Moreover, the shoots grown in the presence of 100 µM Cd^2+^ exhibited significantly higher antioxidant potential in comparison to the control. Our findings indicate that heavy metals are able to stimulate phenolic compound biosynthesis in *Dracocephalum* shoots without any negative impact on their growth. These results could be of significant importance for the medical, nutraceutical and agronomic industries.

## 1. Introduction

Medicinal plants are sources of a wealth for valuable bioactive metabolites and there is growing interest in identifying ways to increase their yield. Particularly rich in polyphenols are species from the *Lamiaceae* family, including those belonging to the genus *Dracocephalum* such as *D. ruyschiana*. This rare medicinal plant migrated from the Russian and Asian steppes to Europe where it is found locally from the boreal regions to the steppes and European mountains [1,2]. *D. ruyschiana* is reported as a traditional medicinal plant in Mongolia. Its aerial parts are used as a natural drug for the treatment of rheumatoid arthritis, laryngitis and acute respiratory infection, headache, diarrhea and gastric ulcers. This plant also possesses antimicrobial, antioxidative, antispasmodic and hepatoprotective effects [3,4]. The aerial parts are known to accumulate many secondary metabolites, including flavonoids and their mono- and tetraglycosides, phenolic acids (chlorogenic acid and caffeoylquinic acids), benzyl alcohol glycosides and essential oil components [4,5].

However, the biosynthesis of bioactive compounds in plants is typically relatively low and variable. Moreover, the population of *D. ruyschiana*, like many other species, is gradually decreasing due to the overexploitation of natural resources. Consequently, alternative methods for producing valuable phytochemicals are being sought. A promising approach to support and enhance the production of bioactive compounds involves in vitro plant cultures [6]. Biotechnological methods not only make cultivation independent of geographical location and seasonal variation but also enable the stimulation of biosynthesis through the controlled regulation of cultivation conditions.

One effective biotechnological strategy is based on elicitation with biotic or abiotic substances [7]. A particularly interesting set of abiotic elicitors are heavy metal ions. Metal ions, such as Ag^+^ and Cd^2+^, act as stressors to induce primary metabolism and accelerate the production of secondary metabolites, especially those with antioxidant activity [8,9]. It has been reported that cadmium-stimulated culture of *Carthamus tinctorius* L. accumulated several times higher flavonoid levels in comparison to a control [10], while titanium dioxide increased flavonoid biosynthesis in *Salvia officinalis* L. up to 50% [11]. In other studies, a large increase in phenolic and anthocyanin contents was observed in *Salvia sclarea* L. exposed to cadmium [12]. Rosmarinic acid (RA) production increased threefold following the addition of vanadyl sulfate to *Lavandula vera* DC. culture [13], and lithospermic acid B increased fourfold in the hairy roots of *Salvia miltiorrhiza* Bunge under the influence of silver thiosulfate [14].

Under stressful conditions, such as heavy metal treatment, plant tissues show elevated levels of reactive oxygen species (ROS) [7,15]. Excessive ROS accumulation can result in oxidative stress, causing DNA, RNA and protein damage and membrane lipid peroxidation. In response to oxidative stress, organisms activate various types of defense mechanisms: these include the increase in activity of antioxidant enzymes such as superoxide dismutase (SOD), peroxidase (POD) and catalase (CAT), and the production of non-enzymatic compounds, such as polyphenols, that bind and neutralize ROS [15].

The aim of the study was to elicit the biosynthesis of polyphenolic compounds in *D. ruyschiana* shoots. For this purpose, the culture was treated with silver and cadmium ions at a concentration of 50 and 100 µM. The level of bioactive compounds accumulated in shoots were determined by HPLC. The study also examines the influence of abiotic stress on the shoot growth, the activity of antioxidant enzymes and the antioxidant potential of the culture.

## 2. Results

### 2.1. Effect of Ag^+^ or Cd^2+^ on Growth and Development of D. ruyschiana Shoot Culture

Nodal segments of in vitro cultivated shoots were used for the propagation of *D. ruyschiana*. The cultures were obtained from the shoot tips of seedlings germinated in vitro from the sterilized seeds. The *D. ruyschiana* shoots were grown in basal MS [16] liquid medium with 0.5 mg/L 6-bezyloamino purine (BAP) and 0.2 mg/L indole-3-acetic acid (IAA). After three weeks of culture, the medium was supplemented with Ag^+^ or Cd^2+^ ions at a concentration of 50 or 100 µM. After another two weeks of cultivation, the parameters of culture growth, development and biochemical response were determined.

**Figure 1:**
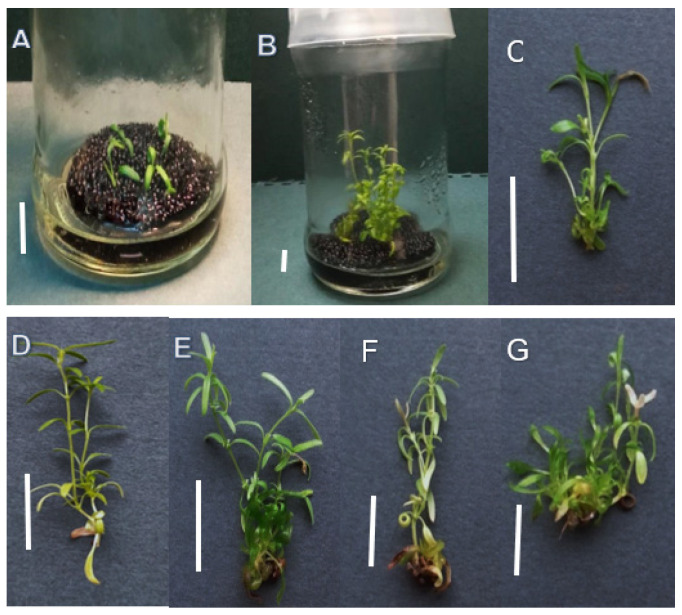
*D. ruyschiana* shoot culture grown in liquid basal MS medium with 0.5 mg/L BAP and 0.2 mg/L IAA; (**A**) inoculum (one day of culture), (**B**) and (**C**) control shoots after five weeks of culture; shoots grown in basal medium with addition of (**D**) 50 µM Ag^+^, (**E**) 100 µM Ag^+^, (**F**) 50 µM Cd^2+^ and (**G**) 100 Cd^2+^ µM after five weeks of culture (two weeks after the application of heavy metal ions). Bar 1 cm.

**Figure 2:**
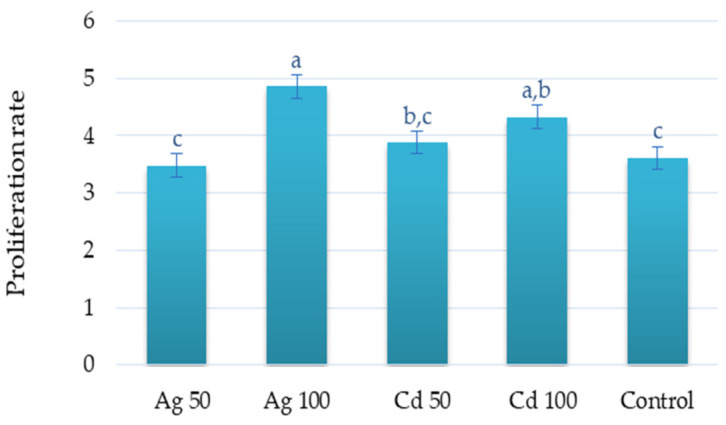
Effect of Ag^+^ and Cd^2+^ at concentrations of 50 or 100 µM on proliferation rate of *D. ruyschiana* culture. The given values represent means ± SD of three independent experimental replicates. Means marked with the same letter were not significantly different (*p* < 0.05).

The highest proliferation rate was observed for the culture exposed to 100 µM of Ag^+^ (4.9 shoot/explant) followed by 100 µM of Cd^2+^ (4.3 shoot/explant) (Figure 1 and Figure 2). No significant differences in shoot regeneration were found between the 50 µM treatments and the control.

**Figure 3:**
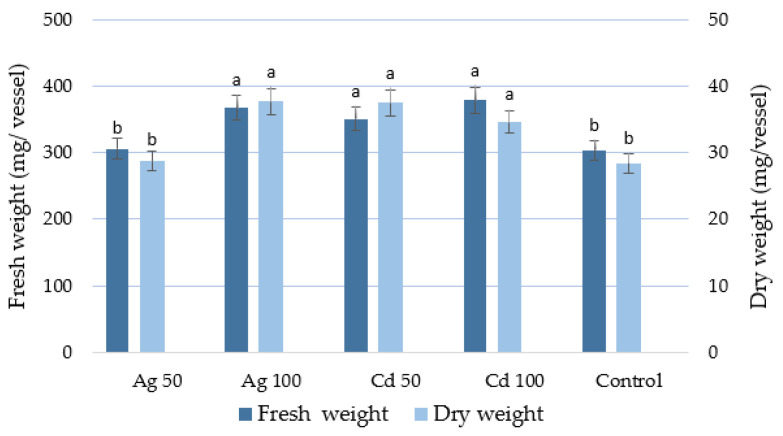
Effect of Ag^+^ and Cd^2+^ at concentrations of 50 or 100 µM on biomass of *D. ruyschiana* culture. The given values represent means ± SD of three independent experimental replicates. Means marked with the same letter were not significantly different (*p* < 0.05).

The treated *D. ruyschiana* culture with the higher concentration of heavy metal ions yielded a significantly larger quantity of fresh biomass than the control; the fresh weight after two weeks of exposure was 367.2–378.5 mg/vessel (Figure 3), i.e., a 60-fold increase compared to the fresh weight of the inoculum. A higher dry weight of the culture was achieved in shoots cultivated in the presence of both metals at 100 µM and Cd^2+^ at 50 µM, i.e., almost 30% higher than the control value (Figure 3).

**Figure 4:**
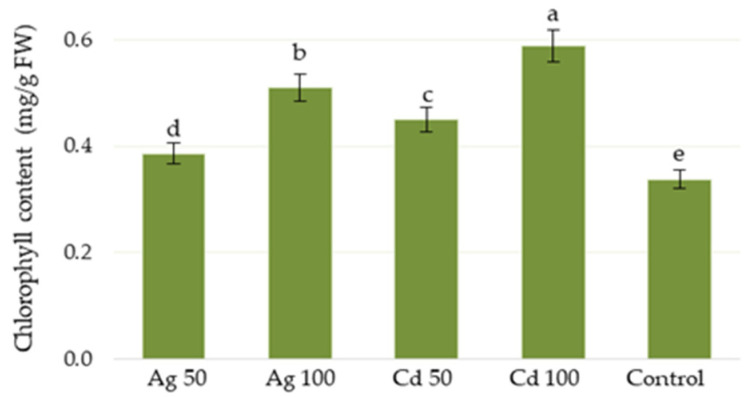
Effect of Ag^+^ or Cd^2+^ at concentrations of 50 or 100 µM on chlorophyll content in *D. ruyschiana* culture. The given values represent means ± SD of three independent experimental replicates. Means marked with the same letter were not significantly different (*p* < 0.05).

The presence of cadmium or silver in culture medium increased the total chlorophyll content in shoots of *D. ruyschiana*, with cadmium stimulating chlorophyll biosynthesis more intensely than silver; in addition, better results were observed at 100 µM than 50 µM (Figure 4). The maximum chlorophyll level was achieved with 100 µM of Cd^2+^ (0.59 mg/g FW), i.e., 75% higher than the control.

### 2.2. Effect of Ag^+^ and Cd^2+^ on Production of Phenolic Compounds in D. ruyschiana Shoot Culture

**Figure 5:**
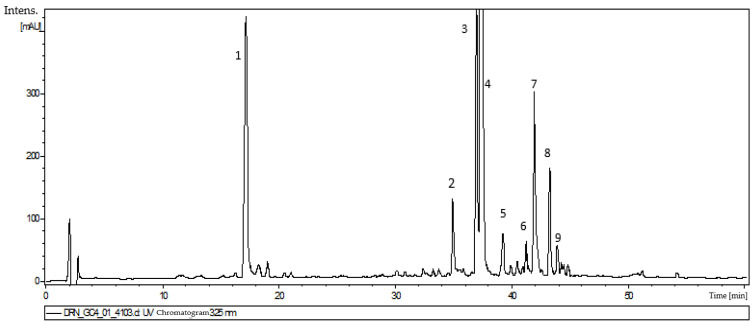
Chromatogram of extract of *D. ruyschiana* shoots grown in MS medium with 0.5 mg/L BAP and 0.2 mg/L IAA: (1) chlorogenic acid, (2) dicaffeoylquinic acid, (3) acacetin rhamnosyl-trihexoside, (4) rosmarinic acid, (5) apigenin caffeoyl-rhamnoside, (6) apigenin *p*-coumaroyl-rhamnoside (I), (7) acacetin acetyl-rhamnosyl-trihexoside, (8) apigenin *p*-coumaroyl-rhamnoside (II) and (9) methyl rosmarinate.

Nine compounds were detected in the 80% methanol extract from *D. ruyschiana* shoots. Four of them, corresponding to peaks 1, 2, 4 and 9 on the UPLC-PDA-ESI-MS chromatogram (Figure 5) were identified by UV-Vis spectra and MS fragmentation patterns as polyphenolic acids. Peak 1 was characterized as chlorogenic acid (CA), Peak 2 as dicaffeoylquinic acid, Peak 4 as rosmarinic acid (RA) and Peak 9 as its methyl ester (Table 1). Identification was performed by comparison with authentic standards and literature data [17,18,19,20].

The remaining five peaks were identified as flavonoid derivatives, with peaks 3 and 7 showing a fragmentation ion [M + H]^+^ at *m*/*z* 285 (in positive mode) assigned to an acacetin derivative [4] (Table 1). Based on the MS fragmentation data and literature data [21,22,23,24], Compound 3 was identified as acacetin rhamnosyl-trihexoside, and 7 as acetyl-rhamnosyl-trihexoside. Peaks 5, 6 and 8, showing a fragmentation ion [M − H]^−^ at *m*/*z* 269 (in negative mode), were identified as representing apigenin derivatives (Table 1). Peak 5 was tentatively assigned to caffeoyl-rhamnoside, and 6 and 8 to *p*-coumaroyl-rhamnosides (I and II).

The levels of individual polyphenolic compounds in shoots of *D. ruyschiana* depended on the presence and concentration of heavy metals added to the medium. The highest total phenolic compound content was found in shoots cultivated on medium supplemented with 100 µM Cd^2+^ (25.9 mg/g DW) (Table 2), with values over twice as high as the control. The addition of the elicitor to the medium stimulated the production of rosmarinic acid and chlorogenic acid most intensely. Treatment with 100 µM Cd^2+^ resulted in levels of 10.95 mg/g DW for RA and 7.66 mg/g DW for CA, with these respective values being about two and three times higher than the control. Interestingly, the RA biosynthesis was more intensely stimulated by lower concentrations of Ag and Cd, with 50 µM Cd^2+^ inducing concentrations as high as 14.72 mg per g DW. However, a higher concentration of cadmium ions enhanced flavonoid production more intensely; with exposure resulting in a twofold increase in total apigenin derivative level (Table 2). The remaining treatments increased the level of apigenin derivatives only slightly or did not change it compared to that obtained for the control. In contrast, the treatments inhibited the biosynthesis of acacetin rhamnosyl-trihexoside; however, 100 µM Cd^2+^ increased the accumulation of acacetin acetyl-rhamnosyl-trihexoside twofold compared to the control (Table 2).

### 2.3. Antioxidant Response of D. ruyschiana Shoots Under Ag^+^ and Cd^2+^ Elicitation

**Figure 6:**
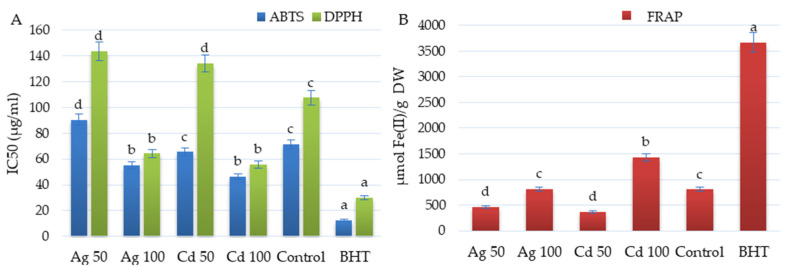
Effect of 50 or 100 µM Ag^+^ and Cd^2+^ on antioxidant activity of the extract from *D. ruyschiana* culture (ABTS, DPPH (**A**) and FRAP (**B**) assays). The given values represent means ± SD of three independent experimental replicates. Means marked with the same letter were not significantly different (*p* < 0.05). Butylated hydroxytoluene (BHT) was used as a positive reference.

The antioxidant activity of the shoots of *D. ruyschiana* was evaluated using DPPH, ABTS and FRAP tests (Figure 6). The strongest antiradical activity was observed for shoots exposed to 100 µM Cd^2+^ and Ag^+^, with respective IC_50_ values of 55.55 and 64.22 µg/mL (DPPH) and IC_50_ of 46.22 and 55.19 µg/mL (ABTS) (Figure 6A). Compared to the untreated shoots, these values were 50% lower following the DPPH test, and 22–35% lower following the ABTS test. Additionally, *D. ruyschiana* shoots exposed to 100 µM Cd^2+^ exhibited nearly a two-fold higher reduction potential following the FRAP test compared to the control shoots (Figure 6B).

**Figure 7:**
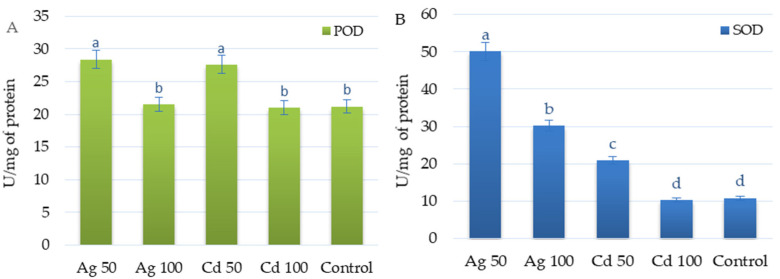
Effect of 50 or 100 µM Ag^+^ and Cd^2+^ on antioxidant enzyme activities: POD (**A**) and SOD (**B**) in *D. ruyschiana* shoots. The given values represent means ± SD of three independent experimental replicates. Means marked with the same letter were not significantly different (*p* < 0.05). POD—peroxidase, SOD—superoxide dismutase.

SOD and POD activity demonstrated different responses to heavy metal exposure. Supplementation with 50 µM Ag^+^ or 50 µM Cd^2+^ had similar effects on POD activity, resulting in a 35% increase in activity compared to non-elicited culture (Figure 7A).

In contrast, silver increased SOD activity much more intensely than cadmium. The SOD activity in shoots exposed to 50 µM Ag^+^ was 2.5 times higher than in those exposed to 50 µM Cd^2+^, and five times higher than in the control (Figure 7B). Increasing the concentration of metals to 100 µM decreased the SOD activity by 1.6 times in the case of silver and two times in the case of cadmium. The SOD level in shoots exposed to 100 µM Cd^2+^ was similar to that recorded in the control.

## 3. Discussion

The impact of heavy metals on plant health is influenced by the metal and its concentration and the sensitivity and resistance of the plant species to metal-induced oxidative stress. Whereas some species demonstrate stunted growth or development in response to heavy metal exposure, others have evolved defense mechanisms. For example, the addition of both cadmium and silver ions at a concentration of 100 μM reduced the biomass of the *Phoenix dactylifera* L. culture by more than twofold [25]. Exposure to cadmium reduced the dry weight of *S. sclarea* shoots by about 15% [14], and exposure to silver ions reduced *Catharanthus roseus* (L.) G. Don. by up to 30% [26]. In contrast, Ag^+^ promoted the biomass of hairy root of *Salvia castanea* Diels. even with a 25% increment over the control [27]. In addition, silver stimulated *Solanum nigrum* shoot proliferation intensely, increasing the number by more than threefold compared to the control; this increase was particularly apparent in the range of 200–600 μM [28]. Moreover, 90 μM Cd^2+^ stimulated the growth of *Arabis paniculata* Franch significantly, and even the highest concentration of ions used in that experiment led to culture growth comparable to that of the control; Cd treatment also did not affect chlorophyll concentrations regardless of the concentration [29]. In contrast, 100 to 200 μM silver increased the chlorophyll level in *Solanum* shoots by more than twofold [30].

In our present study, two concentrations (50 and 100 μM) of cadmium chloride and silver nitrate were used. These values were chosen based on other elicitation experiments that used a wide range of metal concentrations; for example, with cadmium concentrations from 0.5 to 500 μM [14,31,32,33].

Plants can usually manage metal stress up to certain concentrations. The way that growth is stimulated at low doses and inhibited at high doses is referred to as the hormetic dose response [34]. However, these doses may vary greatly between species due to their different sensitivities. For example, cadmium drastically reduced the proliferation of *Albizia lebbeck* (L.) Benth. at a concentration of 5 μM [35], but did not reduce the production of biomass of *Trigonella foenum-graecum* L. at 500 μM [33]. Also, in the present study, the shoots of *D. ruyschiana* showed a high tolerance to Cd^2+^ and Ag^+^ with increased proliferation, chlorophyll level and biomass accumulation, especially at a concentration of 100 μM.

The data suggest that heavy metal tolerance and protection may operate through various modes. One is based on limiting the entry of the metals into the cytoplasm, i.e., by limiting their uptake by the plant or by increasing their accumulation in the cell wall [36]. Plants can also enhance tolerance to heavy metal stress through osmoregulation, which involves the increased production of osmolytes such as sugars and proteins; these act as osmoprotectants, helping to maintain cellular osmotic balance and protect against oxidative damage and metal-induced dehydration. Increased sugar concentrations also enhance plant tolerance to abiotic stress by altering signaling pathways, triggering the production of repair enzymes, and increasing ROS scavenging efficiency [37]. In *C. tinctorius*, exposure to metal, particularly high doses, elevates protein and sugar levels and increased biomass [10]. As such, the accumulation of shoot biomass in contaminated environments can represent a survival strategy by the plant. The plant accumulates sugar by increasing photosynthesis, and the first visible manifestation of this may be an increase in photosynthetic pigment production [10,30]; it was noted in *D. ruyschiana* following heavy metal stimulation, especially at higher metal concentrations and particularly so in the presence of cadmium.

On the other hand, some studies suggest that increased plant growth and proliferation may be also associated with the inhibition of the ethylene molecule, which has a negative effect on the chlorophyll content [38]. Silver nitrate turned out to be a potent inhibitor of ethylene action, blocking or reducing the capacity of its ETR1 receptor [39]. Studies on *Solanum tuberosum* L. indicated that the silver presence prevented the binding of copper, a cofactor required for ethylene activity, resulting in an increase in the total chlorophyll content, especially at high silver concentrations (100–200 μM) [30]. However, Ali et al. [40] proposed that the growth stimulation of *Caralluma tuberculata* N.E.Br. culture observed following exposure to silver could have resulted from enhanced nutrient uptake from the culture medium due to partial damage to the cell wall and increased permeability.

Other physiological adaptations include the secretion of enzymatic and non-enzymatic antioxidant compounds, which reduced the production of ROS and neutralized them [41]. The plant employs various enzymatic antioxidant defense mechanisms [42], including SOD, which converts superoxide radical to H_2_O_2_ and O_2_, and POD for scavenging H_2_O_2_ [43].

Our present findings indicate that *D. ruyschiana* shoots demonstrated elevated POD activity when exposed to cadmium and silver at 50 µM, but not at higher concentrations. In contrast, higher SOD levels induced greater increases in SOD activity, and significantly higher levels were noted in the presence of Ag^+^ than in the presence of Cd^2+^. The possible reason could be associated with the consumption of existing enzyme stock, needed to neutralize the increased levels of free radicals [10,44]. Although antioxidant enzyme activity has generally been reported to increase with metal concentration [40,45], some reports have shown that increased heavy metal stress was associated with lower antioxidant enzyme activity [10,44,46]. This indicates that above certain concentrations, metals could inhibit enzyme systems, for example by damaging and/or deactivating them. It was previously noticed that the activity of POD increased as a response to oxidative stress induced by 50 and 100 µM Cu in the leaves of tomatoes, but broke down at higher metal concentrations [44]. Similarly, SOD activities in *C. tinctorius* increased significantly at 150 µM Cd^2+^, but decreased when the concentration was increased to 200 µM, and CAT activity peaked at 100 µM and decreased at higher concentrations [10].

The activity of antioxidant enzymes could also vary depending on the length of exposure [47]. For example, in *Solanum lycopersicum* L., oxidase activity increased significantly in the first day after copper supplementation, but started to decrease after the second day, and dropped drastically over the next three days. The authors attribute this to metabolism disruption caused by Cu toxicity [44]. It is therefore possible that during the two-week exposure in the present study, the activity of SOD and POD in the *D. ruyschiana* shoots changed, with the final level reflecting their response to the elevated stress caused by the higher concentration of the metals and the higher toxicity of cadmium in comparison to silver.

Non-enzymatic secondary metabolites such as polyphenols also play a supporting role in protecting against ROS. The exogenous application of silver and cadmium ions has been reported to induce the biosynthesis of such compounds as a result of oxidative injury [48,49]. The compounds can form stable complexes with heavy metal ions, thus preventing the development of oxidative stress [50]. Cadmium used at concentrations from 10 to 200 μM increased the level of all flavonoids identified in regenerated shoots of *C. tinctorius* [10], while the amount of chlorogenic acid in *Vaccinium corymbosum* L. increased to 15% following cadmium treatment [51]. Also, the silver ions promoted the biosynthesis of polyphenols in several species; Lam et al. [52] reported that the amount of acacetin and acacetin glucosides in *Agastache rugosa* Kuntze exposed to 100 μM silver nitrate were about 10% higher than in untreated plants, and 50 μM stimulated rosmarinic acid production in *Thymus lotocephalus* G. López and R. shoots by 25% [53].

The influence of heavy metals on the biosynthesis of secondary metabolites in the shoots of *D. ruyschiana* is described in the current study. Some of the phenolic metabolites identified in shoot culture such as chlorogenic acid, acacetin rhamnosyl-trihexoside, acacetin acetyl-rhamnosyl-trihexoside, apigenin *p*-coumaroyl-rhamnoside (II) have previously been detected in aerial parts of this species growing in the field [4,5]. Rosmarinic acid, methyl rosmarinate apigenin caffeoyl-rhamnoside, apigenin p-coumaroyl-rhamnoside and dicaffeoylquinic acid, were detected for the first time in *D. ruyschiana* shoots, but these compounds are known in other *Dracocephalum* species; rosmarinic acid, and methyl rosmarinate were found in aerial parts of *D. moldavica* L., *D. heterophyllum* Benth., *D. foetidum* Bunge. and *D. forrestii* W.W. Smith [54,55,56,57], while the dicaffeoylquinic acid and apigenin caffeoyl–rhamnoside were identified in transformed shoots of *D. forrestii* [24].

In the present study, the metals stimulated a high level of biosynthesis of the predominant compound in the extract, rosmarinic acid, with the strongest effect observed for 50 µM Cd^2+^. This may indicate that rosmarinic acid, the main polyphenol of the plant, performs the main defensive functions in situations of oxidative stress. The levels of chlorogenic acid and flavonoids in *D. ruyschiana* shoots were also modified by heavy metals, with the higher concentration of cadmium having the greatest effect.

The presence of a high polyphenol content has been associated with increased antioxidant potential in elicited cultures of *A. rugosa*, *A. annua* or *C. tinctorius* [12,52,58]. A similar effect was also observed for *D. ruyschiana* shoots, where the extract from shoots exposed to 100 µM Cd^2+^ demonstrated both the strongest antioxidant potential and the highest polyphenol content.

## 4. Materials and Methods

### 4.1. Plant Material

*D. ruyschiana* shoot culture was established from seeds provided by the Innsbruck University Botanical Garden. Surface sterilization of the seeds was performed with 70% ethanol for 10 s and subsequently by 1% (*v*/*v*) sodium hypochlorite for 2 min and three washings with sterilized distilled water. The seeds were placed on Murashige and Skoog (MS) [16] agar (0.7%) medium to produce aseptic seedlings. The apical buds of four-week-old seedlings were placed on the basal MS agar medium with 0.5 mg/L 6-benzylaminopurine (BAP) and 0.2 mg/L indole-3-acetic acid (IAA) for culture establishment. The previous study showed that this type and concentration of phytohormones was optimal for the cultivation of another *Dracocephalum* species [20]. The shoots were subcultured every five weeks.

### 4.2. Culture Conditions

Fragments with a single nodal segment (about 0.5–0.7 cm in length) were taken from the five-week-old shoots of *D. ruyschiana* (from 15–17 subculture) as the explants. Groups of five explants (initial fresh weight about 6.5 mg) were placed into a glass growth vessel with 25 mL liquid MS medium containing BAP (0.5 mg/L) and IAA (0.2 mg/L). In order to avoid the complete submersion of explants in the liquid medium, polyurethane foam (5 cm × 5 cm × 0.7 cm) (EuroFoam, Zgierz, Poland) was placed as support at the bottom of the vessel [59]. Polyurethane foam is inert to plant material, does not absorb medium components, and can be repeatedly sterilized in an autoclave (17 min, 121 °C) without changes to its physical or chemical properties.

After three weeks of culture growth, aqueous solutions of argentum nitricum (AgNO_3_) or cadmium chloride (CdCl_2_) were added into the growth medium using Sterile Syringe Filters (0.22 µm) to a final concentration 50 or 100 μM. The effects of metal ion treatments were evaluated after two consecutive weeks of growth. Shoots cultivated in medium supplemented with distilled water without the addition of heavy metals were used as controls.

The shoots were grown under a 16 h photoperiod (light intensity 50 µM/m^2^/s) at 26 ± 2 °C. The experiment was conducted in three replicates including twenty explants each. After two weeks of exposure to heavy metals, the proliferation rate, i.e., the mean number of new buds (<0.5 cm long) and/or shoots (≥0.5 cm long) on an explant, and their fresh (FW) and dry weight (DW) (mg/growth vessel) were recorded.

### 4.3. Measurement of Chlorophyll Content

The chlorophyll content was determined spectrophotometrically according to the method described by Wellburn [60]. Chlorophyll content was expressed in mg/g FW as the sum of chlorophyll a and chlorophyll b. Absorbance was measured at 664 nm (chlorophyll a) and 647 nm (chlorophyll b).

### 4.4. Shoot Extraction

Lyophilized shoots (100 mg for the phytochemical analysis and 300 mg for the biological assays) were pulverized and extracted three times with 20 mL methanol:water (8:2 *v*/*v*) solution for 15 min in an ultrasonic bath (UD-20 ultrasonic disintegrator; Techpan, Warsaw, Poland). The extracts were combined and evaporated under reduced pressure.

### 4.5. Qualitative UPLC-PDA-ESI-MS Analysis

The compounds present in the extract were identified using UPLC-PDA-ESI-MS using a UPLC-3000 RS apparatus (Dionex, Germering, Germany) with DAD detection and an AmaZon SL ion trap mass spectrometer with an ESI interface (Bruker Daltonik GmbH, Bremen, Germany) with a Zorbax SB-C18 column (150 × 2.1 mm, 1.9 μm) (Agilent, Santa Clara, CA, USA). The mobile phase consisted of 0.1% formic acid in water (A) and 0.1% formic acid in acetonitrile (B). The course and details of the analysis have been described earlier [61]. The metabolites were tentatively identified by comparison of their UV-Vis spectra, mass spectra and the retention times with those for standard compounds and literature data [4,20,24].

### 4.6. Qualitative HPLC Analysis

The samples were dissolved in 2 mL of 80% (*v*/*v*) methanol:water solution and filtered (PTFE syringe filter; 0.22 µm). The phenolic metabolite contents were determined using an Agilent Technologies 1290 Infinity HPLC apparatus (Santa Clara, CA, USA) with a diode array detector (DAD) and Eclipse XDB-C18 column (150 × 4.6 mm, 5 µm). The mobile phase consisted of 0.1% formic acid in acetonitrile (A) and 0.1% formic acid in water (B). The solvent system used for elution was: 0–1 min (95% B); 1–10 min (95–90% B); 10–11 min (90–85% B); 11–40 min (85–80% B); 40–45 min (80–50% B); 45–50 min (50–0% B); 50–53 (0% B). All gradients were linear. The flow rate was 1.6 mL/min. Post-run, the initial phase composition was used for 7 min. To quantify the phenolic compounds, calibration curves were prepared by plotting the peak area of the standard compounds at each level against the concentration of the sample. The experiment used reference standards purchased from Sigma Aldrich (Darmstadt, Germany): 3,5-di-*O*-caffeoylquinic acid, rosmarinic acid, acacetin, apigenin 7-*O*-glucoside and chlorogenic acid. When an authentic standard was not available, the phenolic compounds were quantified according to the calibration curve of an appropriate similar standard: methyl rosmarinate as rosmarinic acid, acacetin glycosides (Compounds 3 and 7) as acacetin, apigenin derivatives (Compounds 5, 6 and 8) as apigenin glucoside. The content of the identified compounds and the total phenolic content, i.e., the sum of all identified phenolics in the sample, were expressed as mg/g DW.

### 4.7. Antioxidant Activity of Extracts

A ferric reducing antioxidant power (FRAP) assay was performed according to Grzegorczyk-Karolak et al. [62]. The samples containing a hydromethanolic extract, water and FRAP reagent were incubated for 30 min at 37 °C. The absorbance was measured at 595 nm. The activity was determined against a standard calibration curve of 0–2000 μM ferrous sulfate. The results were expressed as μM Fe(II)/g of dry weight of the extract.

A DPPH (1,1-diphenyl-2-picrylhydrazyl) assay was carried out according to Grzegorczyk-Karolak and Kiss [63]. Samples containing 2 mL of extract and 2 mL DPPH (0.2 mM solution of DPPH in methanol) were incubated for 30 min in the dark at room temperature. The antiradical activity was indicated at 517 nm.

The ABTS (2,2′-azino-bis (3-ethylbenzothiazoline-6-sulfonic acid)) scavenging properties were measured according to Grzegorczyk-Karolak et al. [62]. Briefly, 2 mL of extracts were mixed with 2 mL of freshly prepared ABTS solution (mixture of 7 mM ABTS and 2.45 mM potassium persulfate). After 10 min of incubation at 25 °C in the dark, the absorbance of the solutions was measured at 735 nm.

For the antiradical tests (DPPH and ABTS), results were expressed as the half-maximal inhibitory concentration (IC_50_) (µg/mL). Butylated hydroxytoluene (BHT) was used as a positive control in all antioxidant assays.

### 4.8. Activity of Antioxidant Enzymes

The fresh shoots (0.5 g) were homogenized in phosphate buffer (pH = 7.5) and EDTA (4 °C) and centrifuged. The obtained supernatant was then tested for sodium dismutase (SOD) and peroxidase (POD) activity. SOD activity was evaluated by adding the extract to the following mixture: phosphate buffer (pH = 7.8), riboflavin, nitro blue tetrazolium (NBT), L-methionine and EDTA [64]. The absorbance of the samples was measured at 560 nm. POD activity was determined by adding the extract to a mixture containing phosphate buffer (pH = 7), guaiacol and hydrogen peroxide, and the absorbance was analyzed at 470 nm [65]. In both cases, the results were expressed as enzyme units per mg of fresh mass.

A UV-1800 spectrophotometer (Beijing Rayleigh Corp., Beijing, China) was used for all spectrophotometric analyses.

### 4.9. Statistical Analysis

Results represent means ± SD (standard deviation) of three independent experimental replicates calculated with Microsoft Excel 2010 (Microsoft Corporation, Redmond, WA, USA). The means were compared using the ANOVA test, followed by Tukey’s *post hoc* test (*p* < 0.05). The statistical analysis was conducted with Statistica 13.1 PL (StatSoft Inc., Krakow, Poland).

## 5. Conclusions

The present study evaluated the effect of the heavy metals Ag^+^ and Cd^2+^ on the proliferation of *D. ruyschiana* shoots and the accumulation of bioactive compounds in the culture. Our findings indicate that the species has a high tolerance to the above metals. No heavy metal treatment inhibited shoot growth, and in some cases, the treatment even stimulated it. This may indicate that the treatment stimulated the biosynthesis of proteins and sugars, which are part of the osmoprotective mechanism. Some treatments also stimulated the production of polyphenols, particularly rosmarinic acid, whose content in samples treated with 50 µM Cd^2+^ was three times that of the control. The production of antioxidant compounds was accompanied by changes in the activity of antioxidant enzymes such as POD and SOD, thus significantly increasing the culture antioxidant potential. In conclusion, the stress response in *D. ruyschiana* is associated with metal-stimulated growth and the accumulation of non-enzymatic and enzymatic antioxidants. Our findings suggest that *D. ruyschiana* uses various strategies to protect against heavy metal stress depending on the type of metal and its concentration. Therefore, further research to clarify the mechanisms of this protection would be advisable.

## Figures and Tables

**Table 1:** MS fragmentation of compounds from extracts of *D. ruyschiana* shoot culture.

Peak Number	Rt [min]	**Ion Mode**		Tentative Assignation
		[M − H]^−^/[M + H]^+^ * (*m*/*z*)	Main Fragments	
1	17	353	191	Chlorogenic acid
2	34.9	515	**353**, 191	Dicaffeoylquinic acid
3	37	917 *	771, 447, **285**	Acacetin rhamnosyl-trihexoside
4	37.8	359	**197**, 179, 161	Rosmarinic acid
5	39.3	577	415, **269**, 161	Apigenin caffeoyl-rhamnoside
6	41.3	561	**397**, 163	Apigenin p-coumaroyl-rhamnoside (I)
7	42.1	959 *	813, 651, 447, **285**	Acacetin acetyl-rhamnosyl-trihexoside
8	43.3	561	415, **397**, 163	Apigenin p-coumaroyl-rhamnoside (II)
9	43.8	373	179	Methyl rosmarinate

Peak numbers refer to those used in Figure 5. * Positive ion mode [M+H]^+^ (*m*/*z*); **in bold**—the most abundant fragmentation ion.

**Table 2:** The effect of heavy metals on the accumulation of phenolic compounds (mg/g DW) in *D. ruyschiana* shoot culture. The shoots were treated with 50 or 100 µM Ag^+^ and Cd^2+^.

Compounds	Treatment				
	Ag^+^ 50 µM	Ag^+^ 100 µM	Cd^2+^ 50 µM	Cd^2+^ 100 µM	Control
Chlorogenic acid	6.54 ± 0.06 ^a^	5.12 ± 0.17 ^b^	4.59 ± 0.07 ^c^	7.66 ± 0.98 ^a^	2.74 ± 0.02 ^d^
Dicaffeoylquinic acid	0.84 ± 0.05 ^b^	0.59 ± 0.01 ^c^	0.54 ± 0.08 ^c^	0.99 ± 0.06 ^a^	0.41 ± 0.04 ^d^
Acacetin rhamnosyl-trihexoside	0.32 ± 0.03 ^c^	0.22 ± 0.01 ^d^	0.29 ± 0.02 ^c^	0.50 ± 0.03 ^b^	0.73 ± 0.06 ^a^
Rosmarinic acid	13.25 ± 0.05 ^b^	8.88 ± 0.22 ^d^	14.72 ± 0.31 ^a^	10.95 ± 0.33 ^c^	5.15 ± 0.14 ^e^
Apigenin caffeoyl-rhamnoside	0.32 ± 0.03 ^b^	0.21 ± 0.03 ^c^	0.27 ± 0.02 ^b,c^	0.43 ± 0.03 ^a^	0.23 ± 0.03 ^c^
Apigenin *p*-coumaroyl-rhamnoside (I)	0.79 ± 0.02 ^c^	0.57 ± 0.01 ^e^	0.63 ± 0.02 ^d^	2.26 ± 0.11 ^a^	1.15 ± 0.04 ^b^
Acacetin acetyl-rhamnosyl-trihexoside	0.75 ± 0.02 ^b^	0.62 ± 0.02 ^c^	0.45 ± 0.02 ^d^	1.14 ± 0.04 ^a^	0.46 ± 0.01 ^d^
Apigenin *p*-coumaroyl-rhamnoside (II)	0.19 ± 0.02 ^a^	0.11 ± 0.02 ^c^	0.14 ± 0.01 ^b^	0.15 ± 0.03 ^ab^	0.11 ± 0.01 ^c^
Methyl rosmarinate	0.93 ± 0.04 ^b^	0.28 ± 0.05 ^c^	1.11 ± 0.15 ^b^	1.82 ± 0.24 ^a^	1.02 ± 0.05 ^b^
Total phenolics	23.93 ± 0.04 ^b^	16.6± 0.06 ^c^	22.81 ± 0.08 ^b^	25.90 ± 0.39 ^a^	12.00 ± 0.40 ^d^

The given values represent means ± SD of three independent experimental replicates. Means marked with the same letter were not significantly different (*p* < 0.05).

## Data Availability

The original results presented in the study are included in the article.

## References

[1] Lazarević P., Lazarević M., Krivošej Z., Stevanović V. (2009). On the distribution of *Dracocephalum ruyschiana* (Lamiaceae) in the Balkan Peninsula. Phytol. Balc..

[2] Kleven O., Endrestøl A., Evju M., Stabbetorp O.E., Westergaard K.B. (2019). SNP discovery in the northern dragonhead *Dracocephalum ruyschiana*. Conserv. Genet. Resour..

[3] Ligaa U. (2006). Medicinal Plants of Mongolia Used in Western and Eastern Medicine.

[4] Selenge E., Murata T., Kobayashi K., Batkhuu J., Yoshizaki F. (2013). Flavone tetraglycosides and benzyl alcohol glycosides from the Mongolian medicinal plant *Dracocephalum ruyschiana*. Nat. Prod..

[5] Okhlopkova Z.M., Razgonova M.P., Pikula K.S., Zakharenko A.M., Piekoszewski W., Manakov Y.A., Ercisli S., Golokhvast K.S. (2022). *Dracocephalum palmatum* S. and *Dracocephalum ruyschiana* L. Originating from Yakutia: A high-resolution mass spectrometric approach for the comprehensive characterization of phenolic compounds. Appl. Sci..

[6] Isah T., Umar S., Mujib A., Sharma M.P., Rajasekharan P.E., Zafar N., Frukh A. (2018). Secondary metabolism of pharmaceuticals in the plant in vitro cultures: Strategies, approaches, and limitations to achieving higher yield. Plant Cell Tissue Organ Cult..

[7] Narayani M., Srivastava S. (2017). Elicitation: A stimulation of stress in in vitro plant cell/tissue cultures for enhancement of secondary metabolite production. Phytochem. Rev..

[8] Khalili M., Hasanloo T., Tabar S.K.K., Rahnama H. (2009). Influence of exogenous salicylic acid on flavonolignans and lipoxygenase activity in the hairy root cultures of *Silybum marianum*. Cell Biol. Inter..

[9] Narula A., Kumar S., Srivastava P.S. (2005). Abiotic metal stress enhances diosgenin yield in *Dioscorea bulbifera* L. cultures. Plant Cell Rep..

[10] Ejaz B., Mujib A., Syeed R., Mamgain J., Malik M.Q., Birat K., Dewir Y.H., Magyar-Tábori K. (2024). Phytocompounds and regulation of flavonoids in in vitro-grown safflower plant tissue by abiotic licitor CdCl_2_. Metabolites.

[11] Ghorbanpour M. (2015). Major essential oil constituents, total phenolics and flavonoids content and antioxidant activity of *Salvia officinalis* plant in response to nano-titanium dioxide. Indian J. Plant Physiol..

[12] Dobrikova A.G., Apostolova E.L., Hanć A., Yotsova E., Borisova P., Sperdouli I., Moustakas M. (2021). Cadmium toxicity in *Salvia sclarea* L.: An integrative response of element uptake, oxidative stress markers, leaf structure and photosynthesis. Ecotoxicol. Environ. Safe.

[13] Georgiev M., Pavlov A., Ilieva M. (2006). Selection of high rosmarinic acid producing *Lavandula vera* MM cell lines. Process Biochem..

[14] Xiao Y., Gao S., Di P., Chen J., Chen W., Zhang L. (2010). Lithospermic acid B is more responsive to silver ions (Ag^+^) than rosmarinic acid in *Salvia miltiorrhiza* hairy root cultures. Biosci. Rep..

[15] Kruk J., Aboul-Enein B.H., Duchnik E., Marchlewicz M. (2022). Antioxidative properties of phenolic compounds and their effect on oxidative stress induced by severe physical exercise. J. Physiol. Sci..

[16] Murashige T., Skoog F. (1962). A revised medium for rapid growth and bioassay with tobacco tissue culture. Physiol. Plant..

[17] Chen F., Long X., Liu Z., Shao H., Lu L. (2014). Analysis of phenolic acids of Jerusalem Artichoke (*Helianthus tuberosus* L.) responding to salt-stress by liquid chromatography/tandem mass spectrometry. Sci. World J..

[18] Catarino M.D., Silva A.M.S., Saraiva S.C., Sobral A.J.F.N., Cardoso S.M. (2018). Characterization of phenolic constituents and evaluation of antioxidant properties of leaves and stems of *Eriocephalus africanus*. Arab. J. Chem..

[19] Ncube N., Mhlongo M.I., Piater L.A., Steenkamp P.A., Dubery I.A., Madala N.E. (2014). Analyses of chlorogenic acids and related cinnamic acid derivatives from *Nicotiana tabacum* tissues with the aid of UPLC-QTOF-MS/MS based on the in-source collision induced dissociation method. Chem. Cent. J..

[20] Weremczuk-Jeżyna I., Kuźma Ł., Kiss A.K., Grzegorczyk-Karolak I. (2018). Effect of cytokinins on shoots proliferation and rosmarinic and salvianolic acid B production in shoot culture of *Dracocephalum forrestii* W. W. Smith. Acta Physiol. Plant..

[21] Lin L.L., Harmly J.M. (2010). Identification of the phenolic components of chrysanthemum flowers (*Chrysanthemum morifolium* Ramat). Food Chem..

[22] Bakr R.O., El Bishbishy M.H. (2016). Profile of bioactive compounds of *Capparis spinosa* var. *aegyptiaca* growing in Egypt. Rev. Bras. Farmacogn..

[23] Wu W., Liu Z., Song F., Lin S. (2004). Structural analysis of selected characteristic flavones by electrospray tandem mass spectrometry. Anal. Sci..

[24] Weremczuk-Jeżyna I., Skała E., Kuźma Ł., Kiss A.K., Grzegorczyk-Karolak I. (2019). The effect of purine-type cytokinin on the proliferation and production of phenolic compounds in transformed shoots of *Dracocephalum forrestii*. J. Biotechnol..

[25] Al-Khayri J.M., Poornananda M.N. (2020). Elicitor-induced production of biomass and pharmaceutical phenolic compounds in cell suspension culture of date palm (*Phoenix dactylifera* L.). Molecules.

[26] Paeizi M., Karimi M., Razavi K. (2018). Changes in medicinal alkaloids production and expression of related regulatory and biosynthetic genes in response to silver nitrate combined with methyl jasmonate in *Catharanthus roseus in vitro* propagated shoots. Plant Physiol. Biochem..

[27] Li B., Wang B., Li H., Peng L., Ru M., Liang Z., Zhu Y. (2016). Establishment of *Salvia castanea* Diels *f. tomentosa* Stib. hairy root cultures and the promotion of tanshinone accumulation and gene expression with Ag+, methyl jasmonate, and yeast extract elicitation. Protoplasma.

[28] Geetha G., Harathi K., Naidu C.V. (2016). Role of silver nitrate on flowering and shoot regeneration of *Solanum* nigrum—Important multipurpose medicinal plant. Am. J. Plant Sci..

[29] Qiu R.L., Zhao X., Tang Y.T., Yu F.M., Hu P.J. (2008). Antioxidative response to Cd in a newly discovered cadmium hyperaccumulator, *Arabis paniculata* F. Chemosphere.

[30] Rostami F., Ehsanpour A. (2010). The effect of silver thiosulfate (STS) on chlorophyll content and the antioxidant. J. Cell Mol. Res..

[31] Wiszniewska A., Hanus-Fajerska E., Muszyńska E., Smoleń S. (2017). Comparative assessment of response to cadmium in heavy metal-tolerant shrubs cultured in vitro. Water Air Soil Pollut..

[32] Açıkgoz M.A. (2020). Establishment of cell suspension cultures of *Ocimum basilicum* L. and enhanced production of pharmaceutical active ingredients. Ind. Crop. Prod..

[33] De D., De B. (2022). Elicitation of diosgenin production in *Trigonella foenum-graecum* L. seedlings by heavy metals and signaling molecules. Acta Physiol. Plant..

[34] Calabrese E.J., Blain R. (2009). Hormesis and plant biology. Environ. Pollut..

[35] Perveen S., Anis M., Aref I.M. (2012). In vitro morphogenic response and metal accumulation in *Albizia lebbeck* (L.) cultures grown under metal stress. Eur. J. For. Res..

[36] Atabayeva S.D., Minocha R., Minocha S.C., Rakhymgozhina A., Nabieva A.M., Nurmahanova A.C., Kenzhebayeva S.S., Alybayeva R.A., Asrandina S.S. (2020). Response of plants to cadmium stress. Int. J. Biol. Chem..

[37] Gugale G.S., Bhusare B.P., Ambawade M.S., Kshatriya A.S., Barwant M.M., Mhaske A.K. (2021). Effect of cadmium chloride on seed germination, seedling growth parameters, and proline content in maize (*Zea mays*). Int. J. Res. Educ. Sci. Meth..

[38] Bora G., Gogoi H.K., Handique P.J. (2019). Influence of silver nitrate and glutamine on in vitro organogenesis of Lota Bhot (*Capsicum chinense* Jacq.), an indigenous pungent pepper variety of Assam. J. Appl. Biol. Biotechnol..

[39] Zhao X.C., Qu X., Mathwes D.E., Schaller G.E. (2002). Effect of ethylene-pathway mutations upon expression of the ethylene receptor ETR1 from *Arabidopsis*. Plant Physiol..

[40] Ali A., Mohammad S., Khan M.A., Raja N.I., Arif M., Kamil A., Mashwani Z.U.R. (2019). Silver nanoparticles elicited in vitro callus cultures for accumulation of biomass and secondary metabolites in *Caralluma tuberculata*. Artif. Cells Nanomed. Biotechnol..

[41] Hall J.L. (2002). Cellular mechanisms for heavy metal detoxication and tolerance. J. Exp. Bot..

[42] Kisa D., Elmastaş M., Öztürk L., Kayır Ö. (2016). Responses of the phenolic compounds of *Zea mays* under heavy metal stress. Appl. Biol. Chem..

[43] Zhang S., Zhang H., Qin R., Jiang W., Liu D. (2009). Cadmium induction of lipid peroxidation and effects on root tip cells and antioxidant enzyme activities in *Vicia faba* L.. Ecotoxicology.

[44] Martins L.L., Mourato M.P. (2006). Effect of excess copper on tomato plants: Growth parameters, enzyme activities, chlorophyll, and mineral content. J. Plant Nutr..

[45] Irfan M., Hayats S., Ahmed A., Alyemeni M.N. (2013). Soil cadmium enrichment: Allocation and plant physiological manifestations. Saudi J. Biol. Sci..

[46] Hassan M.J., Raza M.A., Rehman S.U., Ansar M., Gitari H., Khan I., Wajid M., Ahmed M., Shah G.A., Peng Y. (2020). Effect of cadmium toxicity on growth, oxidative damage, antioxidant defense system and cadmium accumulation in two orghum cultivars. Plants.

[47] Carmen E.M., Souza V., Bucio L., Hernández E., Damián-Matsumura P., Zaga V., Gutiérrez-Ruiz M.C. (2002). Cadmium induces alpha(1)collagen (I) and metallothionein II gene and alters the antioxidant system in rat hepatic stellate cells. Toxicology.

[48] Anjitha K.S., Sameena P.P., Puthur J.T. (2021). Functional aspects of plant secondary metabolites in metal stress tolerance and their importance in pharmacology. Plant Stress.

[49] Bhaduri A.M., Fulekar M.H. (2012). Antioxidant enzyme responses of plants to heavy metal stress. Rev. Environ. Sci. Biotechnol..

[50] Jiao Z., Shi Y., Wang J., Wang Z., Zhang X., Jia X., Du Q., Niu J., Liu B., Du R. (2023). Integration of transcriptome and metabolome analyses reveals sorghum roots responding to cadmium stress through regulation of the flavonoid biosynthesis pathway. Front. Plant Sci..

[51] Manquián-Cerda K., Escudey M., Zúñiga G., Arancibia-Miranda N., Molina M., Cruces E. (2016). Effect of cadmium on phenolic compounds, antioxidant enzyme activity and oxidative stress in blueberry (*Vaccinium corymbosum* L.) plantlets grown in vitro. Ecotoxicol. Environ. Saf..

[52] Lam V.P., Beomseon L., Anh V.K., Loi D.N., Kim S., Kwang-Ya L., Park J. (2023). Effectiveness of silver nitrate application on plant growth and bioactive compounds in *Agastache rugosa* (Fisch. & CA Mey.) Kuntze. Heliyon.

[53] Gonçalves S., Mansinhos I., Rodríguez-Solana R., Pérez-Santín E., Coelho N., Romano A. (2019). Elicitation improves rosmarinic acid content and antioxidant activity in *Thymus lotocephalus* shoot cultures. Ind. Crop. Prod..

[54] Zhang J.I., Yan R.J., Yu N., Zhang X., Chen D.J., Wu T., Xin J.G. (2018). A new caffeic acid tetramer from the *Dracocephalum moldavica* L.. Nat. Prod. Res..

[55] Selenge E., Murata T., Tanaka S., Sasaki K., Batkhuu J., Yoshizaki F. (2014). Monoterpene glycosides, phenylpropanoids, and acacetin glycosides from *Dracocephalum foetidum*. Phytochemistry.

[56] Dang J., Wang S., Shao Y. (2018). Preparative isolation of antioxidative compounds from *Dracocephalum heterophyllum* using off-line two-dimensional reversed-phase liquid chromatography/hydrophilic interaction chromatography guided by on-line HPLC-DPPH assay. J. Chromatogr. B.

[57] Li S.M., Yang X.W., Li Y.L., Shen Y.H., Feng L., Wang Y.H., Zeng H.W., Liu X.H., Zhang C.S., Long C.L. (2004). Chemical constituents of *Dracocephalum forestii*. Planta Med..

[58] Darki B.S., Shabani L., Pourvaez R., Ghannadian S.M. (2019). Effects of CuSO4 and AgNO_3_ on artemisinin and phenolic compound in shoot cultures of *Artemisia annua* L.. J. Plant Process Funct..

[59] Grzegorczyk-Karolak I., Rytczak P., Bielecki S., Wysokińska H. (2017). The influence of liquid systems for shoot multiplication, secondary metabolite production and plant regeneration of *Scutellaria alpina*. Plant. Cell Tiss. Organ Cult..

[60] Wellburn A.R. (1994). The spectra determination of chlorophylls a and b as well as total carotenoids, using various solvents with spectrophotometers of different resolution. J. Plant Physiol..

[61] Grzegorczyk-Karolak I., Ejsmont W., Kiss A.K., Tabaka P., Starbała W., Krzemińska M. (2024). Improvement of bioactive polyphenol accumulation in callus of *Salvia atropatana* Bunge. Molecules.

[62] Grzegorczyk-Karolak I., Kuźma Ł., Wysokińska H. (2015). The effect of cytokinins on shoot proliferation, secondary metabolite production and antioxidant potential in shoot cultures of *Scutellaria alpina*. Plant Cell Tiss. Organ Cult..

[63] Grzegorczyk-Karolak I., Kiss A. (2018). Determination of the phenolic profile and antioxidant properties of *Salvia viridis* L. shoots: A comparison of aqueous and hydroethanolic extracts. Molecules.

[64] Van Rossun M.W.P.C., Alberda M., Van Der Plas L.H.W. (1997). Role of oxidative damage in tulip bulb scale micropropagation. Plant Sci..

[65] Hemeda H.M., Klein B.P. (1990). Effects of naturally occurring antioxidants on peroxidase activity of vegetable extracts. J. Food Sci..

